# Relationship between immune checkpoint proteins, tumour microenvironment characteristics, and prognosis in primary operable colorectal cancer

**DOI:** 10.1002/cjp2.193

**Published:** 2020-12-18

**Authors:** Sara SF Al‐Badran, Lauren Grant, Maejoy V Campo, Jitwadee Inthagard, Kathryn Pennel, Jean Quinn, Prakash Konanahalli, Liam Hayman, Paul G Horgan, Donald C McMillan, Campbell SD Roxburgh, Antonia Roseweir, James H Park, Joanne Edwards

**Affiliations:** ^1^ Academic Unit of Surgery, School of Medicine University of Glasgow, Glasgow Royal Infirmary Glasgow UK; ^2^ Unit of Experimental Therapeutics Institute of Cancer Sciences, Wolfson‐Wohl Cancer Research Centre Glasgow UK; ^3^ Department of Pathology, Queen Elizabeth University Hospital Glasgow UK

**Keywords:** immune checkpoint, TIM‐3, LAG‐3, PD‐1, stromal immune cells, prognosis, colorectal cancer, tumour microenvironment

## Abstract

The tumour microenvironment is an important factor for colorectal cancer prognosis, affecting the patient's immune response. Immune checkpoints, which regulate the immune functions of lymphocytes, may provide prognostic power. This study aimed to investigate the prognostic value of the immune checkpoints TIM‐3, LAG‐3 and PD‐1 in patients with stage I–III colorectal cancer. Immunohistochemistry was employed to detect TIM‐3, LAG‐3, PD‐1 and PD‐L1 in 773 patients with stage I–III colorectal cancer. Immune checkpoint protein expression was assessed in tumour cells using the weighted histoscore, and in immune cells within the stroma using point counting. Scores were analysed for associations with survival and clinical factors. High tumoural LAG‐3 (hazard ratio [HR] 1.45 95% confidence interval [CI] 1.00–2.09, *p* = 0.049) and PD‐1 (HR 1.34 95% CI 1.00–1.78, *p* = 0.047) associated with poor survival, whereas high TIM‐3 (HR 0.60 95% CI 0.42–0.84, *p* = 0.003), LAG‐3 (HR 0.58 95% CI 0.40–0.87, *p* = 0.006) and PD‐1 (HR 0.65 95% CI 0.49–0.86, *p* = 0.002) on immune cells within the stroma associated with improved survival, while PD‐L1 in the tumour (*p* = 0.487) or the immune cells within the stroma (*p* = 0.298) was not associated with survival. Furthermore, immune cell LAG‐3 was independently associated with survival (*p* = 0.017). Checkpoint expression scores on stromal immune cells were combined into a Combined Immune Checkpoint Stromal Score (CICSS), where CICSS 3 denoted all high, CICSS 2 denoted any two high, and CICSS 1 denoted other combinations. CICSS 3 was associated with improved patient survival (HR 0.57 95% CI 0.42–0.78, *p* = 0.001). The results suggest that individual and combined high expression of TIM‐3, LAG‐3, and PD‐1 on stromal immune cells are associated with better colorectal cancer prognosis, suggesting there is added value to investigating multiple immune checkpoints simultaneously.

## Introduction

Colorectal cancer (CRC) contributes to about 10% of reported cancer deaths worldwide [1]. Various factors lead to CRC, including life‐style choices, inherited genetics such as Lynch syndrome (hereditary non‐polyposis colorectal cancer), and pre‐existing chronic conditions including inflammatory bowel disease [2, 3, 4, 5]. CRC staging is rapidly evolving from relying on radiological and pathological assessment of the tumour to recognising the importance of both molecular and tumour microenvironment (TME) characteristics in determining patient survival and treatment response [6, 7, 8, 9, 10, 11]. Additionally, emerging evidence supports the use of markers of systemic inflammation prognostically and predictively in advanced disease [12].

For instance, increased immune response local to the tumour has been shown to be provoked by neoantigen‐bearing mismatch repair (MMR) deficient tumours, as evidenced by increased immune cell density [13, 14, 15]. However, characteristics of the TME are not solely dependent on underlying tumour molecular characteristics, and the prognostic value of the local inflammatory response independent of MMR status is increasingly recognised [16, 17, 18].

Indeed, immune‐mediated inflammation, whether systemic or local, is of prognostic value in CRC. However, whereas systemic inflammation, which is directly related to poor prognosis is easily measured, TME inflammation is more complicated [19], due to the immune system's dual role in cancer [20]. Hence, understanding immune regulation within the TME is imperative in understanding CRC prognosis.

Immune checkpoints are membrane proteins that regulate immune cells by either activating or inhibiting their immune functions [21] and have the potential of acting as prognostic biomarkers. Inhibitory checkpoints like programmed cell death protein‐1 (PD‐1), T‐cell immunoglobulin mucin‐domain containing‐3 (TIM‐3), and lymphocyte‐activation gene 3 (LAG‐3), are all present on T‐cells and all bind to ligands that are secreted by tumour cells [22]. PD‐1, through its interaction with its ligand PD‐L1, aids tumours in evading anti‐tumour immunity. Hence, blocking this interaction is the aim of many therapeutic interventions [23]. However, intrinsic resistance remains an issue. It has been reported that other inhibitory immune checkpoints like TIM‐3 and LAG‐3 become up‐regulated during PD‐1/PD‐L1 blockage, to resist treatment and allow cancer to progress [22, 24]. Hence, combined therapies targeting two or more of these inhibitory checkpoints are being extensively investigated in various stages of pre‐clinical and clinical studies [25, 26, 27].

Since these immune checkpoints are of high therapeutic value, investigating whether their assessment in the TME holds prognostic significance is of great interest. Therefore, the following study aimed to examine the prognostic value of PD‐1, PD‐L1, TIM‐3 and LAG‐3 expression in colorectal tumours and their microenvironment in samples from patients who underwent curative resections of stage I–III CRC. This expression was analysed in relation to patient, tumour, and TME characteristics, systemic inflammation, and cancer‐specific survival.

## Patients and methods

### Patient characteristics and ethics statement

1009 stage I–III patients that had primary operable CRC and underwent curative tumour resection surgery in Glasgow Royal Infirmary, Western Infirmary, or Stobhill Hospitals (Glasgow, UK) between 1997 and 2007 were included in the study. Patients who died within 30 days of surgery or underwent neo‐adjuvant therapy were excluded. Previously constructed tumour microarrays (TMAs) were utilised; four 0.6 mm cores were available per patient to combat tumour heterogeneity. Tumour staging was carried out using the 5th Edition of the AJCC/UICC‐TNM staging system. All patients were followed up for at least five years post resection. This study was approved by the West of Scotland Research Ethics Committee (16/WS/0207) and patient information is held within the Glasgow and Clyde Safe Haven (12/WS/0142).

### Clinicopathological characteristics

Patient age, sex, adjuvant therapy status, tumour site, staging, necrosis, venous invasion and resection margin involvement were obtained from clinical records. Ki67 proliferation index was calculated as previously described [28]. Tumour budding was assessed as previously described within 10 high power fields [29]. MMR status was previously determined for this cohort [16]. MMR proteins MLH1, PMS2, MSH2 and MSH6 were assessed by IHC, and scored deficient when there was loss of staining of at least one MMR protein with normal expression within adjacent epithelium or intra‐tumoural immune cells.

### TME characteristics

Tumour stroma percentage (TSP) was previously determined for this cohort [11]. Briefly, stromal percentage was measured and was graded as low (≤50%) or high (>50%). Inflammatory infiltrate was quantified using the Klintrup–Mäkinen score as low‐grade (no or mild increase in inflammation), and high grade (prominent increase in inflammation with cancer cell destruction) as previously described [30]. The Glasgow Microenvironment Score (GMS) was constructed by combining the TSP and the Klintup–Mäkinen as previously described [31]. CD3^+^, CD8^+^ and FoxP3^+^ immune cell density was assessed using an immunohistochemical method, which included dewaxing and rehydration of cores through graded alcohols, followed by antigen retrieval in Tris–EDTA buffer at pH 8 under pressure, then blocking of peroxidases and non‐specific binding using 3% H_2_O_2_ and 5% horse serum respectively. Cores were incubated overnight at 4 °C in anti‐CD3 at 1:500 (ThermoFisher, Renfrew, UK), anti‐CD8 at 1:100 (Dako, Copenhagen, Denmark), and anti‐FoxP3 at 1:400 (Abcam, Cambridge, MA, USA). Following a 30‐min secondary antibody incubation (ImPRESS, Vector Laboratories, Upper Heyford, UK), ImPACT DAB (Vector Laboratories) was added as chromogen and the slides were then counterstained with haematoxylin, dehydrated and mounted with coverslips. Positive immune cells were counted withing the cancer cell nest and stroma separately.

### Systemic inflammation characteristics

Pre‐operative C‐reactive protein (CRP) and albumin concentrations were recorded prospectively within 30 days of surgery. CRP >10 mg/l and albumin <35 g/l were considered abnormal, and the modified Glasgow Prognostic Score (mGPS) utilised these as previously described [32]. The neutrophil‐to‐lymphocyte ratio (NLR) was previously determined for this cohort utilising a threshold of <3/3–5/>5 [33].

### Immunohistochemistry and visualisation

TMAs were dewaxed in Histoclear (National Diagnostics, CA, USA) followed by rehydration through a decreasing gradient of ethanol. Heat‐induced antigen retrieval was carried out under pressure in a microwave using Tris–EDTA Buffer at pH 9 for anti‐TIM‐3, and citrate buffer at pH 6 for anti‐PD‐1, anti‐LAG‐3 and anti‐PD‐L1. Endogenous peroxidase activity was blocked in 3% H_2_O_2_. Non‐specific antibody binding was blocked using 10% casein (Vector Laboratories) for anti‐TIM‐3 and anti‐LAG‐3 and 5% horse serum (Vector Laboratories) for anti‐PD‐1 and anti‐PD‐L1. TMAs were stained with anti‐TIM‐3 (Cat. TA355031, OriGene, Rockville, MD, USA) at 1:1000 for 30 min at RT, anti‐PD1 (Product No.: HPA035981, Atlas Antibodies, Bromma, Sweden) at 1:100, anti‐PD‐L1 (Cat. 17952‐1‐AP, Proteintech, Rosemont, IL, USA) at 1:100 and anti‐LAG‐3 (Cat.: HPA013967, Sigma‐Aldrich, Gillingham, UK) at 1:1,250, overnight at 4 °C. TMAs were incubated for 30 min at RT in ImmPress (TIM‐3/LAG‐3; Vector Laboratories) or EnVision (PD‐1 and PD‐L1; Dako) and were visualised with ImmPact 3,3′‐diaminobenzidine substrate (Vector Laboratories). Samples were then counterstained in Harris Haematoxylin (ThermoFisher) dehydrated in increasing ethanol gradients and Histoclear, before mounting with Omnimount (National Diagnostics). TMAs were scanned using Hamamatsu NanoZoomer Digital Slide Scanner and visualised in SlidePath (Version 4.0.9, Leica Biosystems, Newcastle, UK). Negative control slides (no antibody, isotype‐matched antibody) were included to rule out nonspecific staining. To ensure the positive staining was specific, full section slides of colorectal cancer or liver tissue were included as known positive controls, as well as cell pellets (HT29) known to have positive expression (see supplementary material, Figure S1).

### Scoring methods

**Figure 1:**
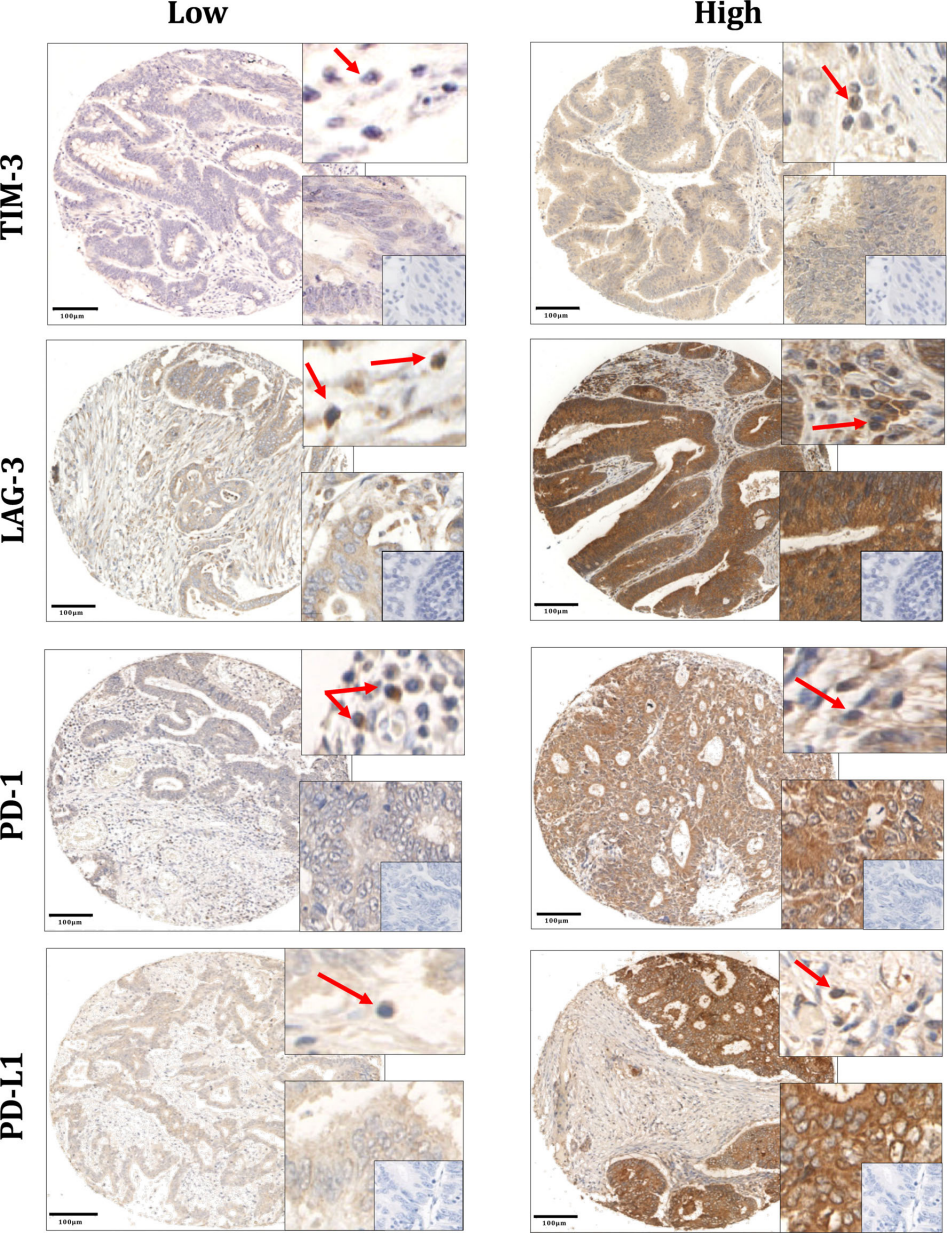
Immune checkpoint expression in colorectal cancer. TIM‐3, LAG‐3, PD‐1 and PD‐L1 showed cytoplasmic staining. For each marker, the top right boxes show examples of positive lymphocytes in the stroma (red arrows); and the bottom right boxes show positive tumour cells at a higher magnification with no‐antibody negative controls within (insets). Scale bars at 100 μm.

Scoring was performed by a single observer blinded to the clinical data (SSFA for TIM‐3, MVC for LAG‐3 and LG for PD‐1 and PD‐L1). To ensure consistency, 10% of cores for each marker was co‐scored by a second observer (JE for TIM‐3 and LAG‐3 and JHP for PD‐1 and PD‐L1). Tumour cell expression was assessed using the weighted Histoscore method [34]. The weighted Histoscore was calculated as follows: (% of unstained tumour cells × 0) + (% of weakly stained tumour cells × 1) + (% of moderately stained tumour cells × 2) + (% of strongly stained tumour cells × 3) to give a range from 0 to 300. All four cores were scored separately, and an average score was taken. Immune cells within the stroma expressing each of the markers were assessed using a total point count of positive lymphocytes for each core (Figure 1). Positive lymphocytes were recognised as brown membrane and/or cytoplasmic stain with blue nuclei. Values from four cores were averaged for each patient.

To ensure reliability and objectivity, 10% of cores were scored digitally on QuPath [35]. In brief, after using the TMA Dearrayer function to create a TMA grid with cores in their correct positions, stain vectors were estimated during pre‐processing by the visual stain editor available in QuPath, to increase staining quality. Then, cells were detected using a watershed cell detection method, and annotations were made to allow QuPath to recognise a variety of tissue types. These included tumour and stroma. Then, a random trees classifier was trained using over 40 features such as perimeter, area, and optical density. Three intensity thresholds were used to represent negative, weak, moderate and strong staining, and after the classifier was built, the auto‐update feature was used to re‐validate the classifier's accuracy in real‐time. The classifier was then saved and applied to all TMA slides that were subjected to QuPath analysis.

### Statistical analysis

Interclass correlation coefficient (ICCC) was used to ensure consistency and objectivity between the main scorer and the co‐scorers and QuPath. Values above 0.75 are indicative of good reliability [36]. Immune checkpoint expression in both the tumour and lymphocytes within the stroma was categorised as either ‘low’ or ‘high’ utilising cut‐offs determined using a receiver operating characteristic (ROC) curve or median. ROC curves were used for tumoural TIM‐3 (132), LAG‐3 (161), PD‐1 (12) and PD‐L1 (139) as well as TIM‐3 (17), PD‐1 (1.6) and PD‐L1 (4.6) expressed on stromal immune cells, while the median was used for stromal immune cells expressing LAG‐3. Associations between immune checkpoint expression and clinicopathological characteristics were assessed using χ^2^‐test. Immune checkpoint expression and cancer‐specific survival was assessed using Kaplan–Meier survival curves with Log‐Rank testing. Univariate Cox regression survival analysis was used to determine hazard ratios (HRs) and 95% confidence intervals (CIs). Multivariable Cox regression survival analysis using a backward conditional elimination model and a statistical significance threshold of 0.05 was performed to identify independent prognostic biomarkers. Due to the number of comparisons of clinicopathological characteristics performed, a Bonferroni corrected *P* value 0.002 (0.05/22) was considered statistically significant for χ^2^‐tests. Otherwise, *P* values 0.05 were considered significant. All statistical analysis was performed using IBM SPSS Statistics Version 24 and was two‐sided.

## Results

Of the 1009 patients, 773 with stage I–III CRC were eligible and had adequate tissue to be included in the analysis (see supplementary material, Figure S2); 528 (68%) patients were over 65 years of age, with 395 (51%) being male. 107 (14%) patients were TNM‐I, 373 (48%) were TNM‐II and 293 were TNM‐III (38%). Of this cohort, MMR data was valid for 761 patients, 133 of which were MMR‐deficient (17%) and 628 of which were MMR‐competent (83%; see supplementary material, Table S4). The median follow‐up was 100 months with 222 cancer deaths. 403 and 457 patients had valid scores for tumour and stromal immune cells TIM‐3 respectively, 413 and 387 had valid scores for tumour and stromal immune cells LAG‐3, 722 and 719 patients had valid scores for tumour and stromal immune cells PD‐1, and 708 had valid scores for tumour and stromal immune cells PD‐L1 (see supplementary material, Figure S2).

QuPath was used to score 10% of cores to ensure reliability and objectivity of staining assessment. The ICCC value of manual scores and QuPath scores was 0.827 for TIM‐3, 0.756 for LAG‐3, 0.842 for PD‐1 and 0.795 for PD‐L1.

### Immune checkpoint expression is associated with cancer‐specific survival

**Figure 2:**
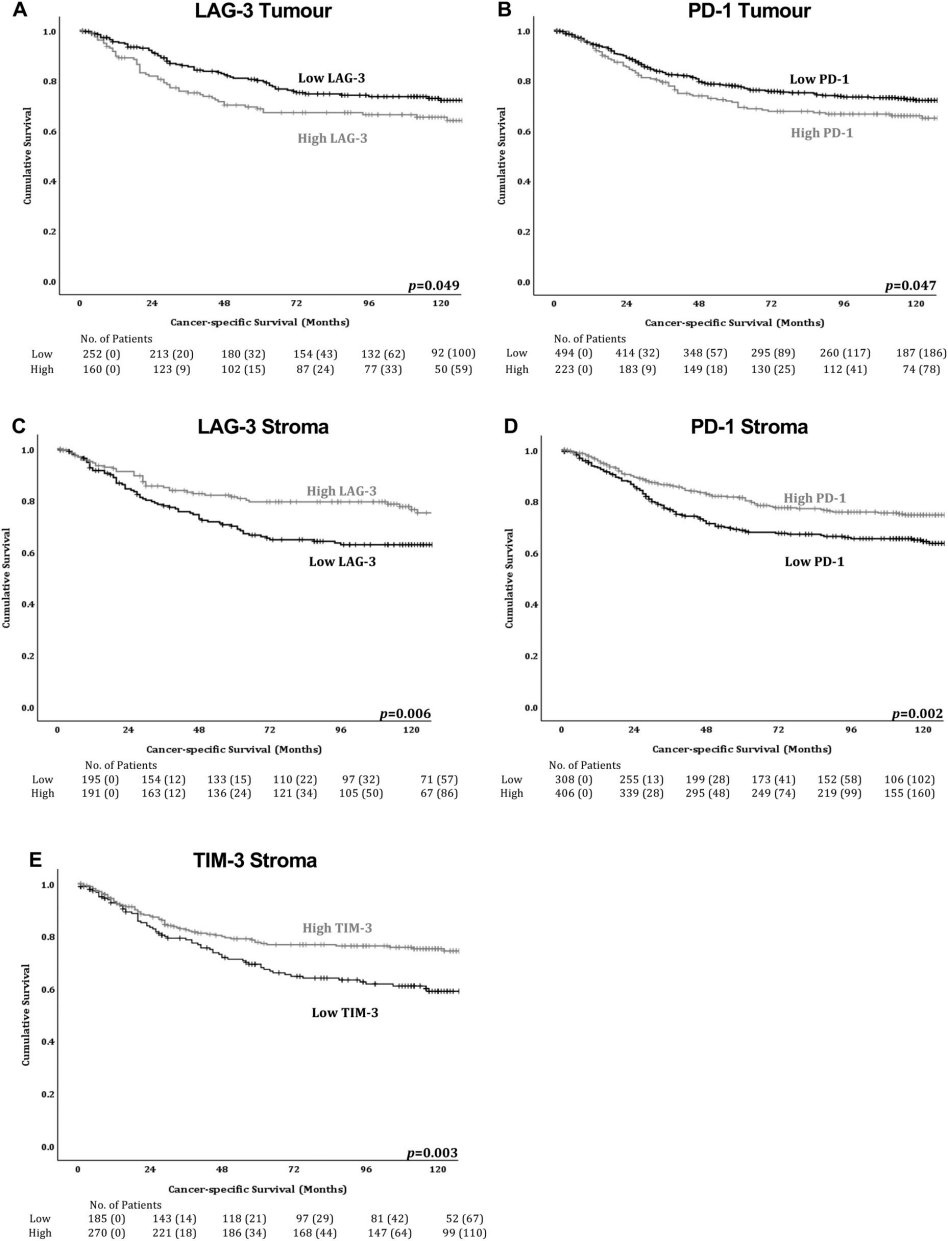
Immune checkpoint expression in the tumour and stromal immune cells of colorectal cancer patients is significantly associated with survival. Kaplan–Meier survival curves for associations between cancer‐specific survival and (A) tumoural LAG‐3, (B) tumoural PD‐1, (C) stromal immune cell LAG‐3, (D) stromal immune cell PD‐1 and (E) stromal immune cell TIM‐3 expression.

Tumoural expression refers to tumour cell expression and stromal immune cell expression refers to expression on immune cells within the stroma. Table 1 shows the association between immune checkpoint expression and cancer‐specific survival. Tumoural TIM‐ 3 expression was not associated with survival (*p* = 0.188), and neither was PD‐L1 in the tumour (*p* = 0.487) or lymphocytes in the stroma (*p* = 0.298). However, high tumoural LAG‐3 (HR 1.45, 95% CI 1.00–2.09, *p* = 0.049; Figure 2A) and PD‐1 (HR 1.34, 95% CI 1.00–1.78, *p* = 0.047; Figure 2B) were significantly associated with poor cancer‐specific survival, whereas high stromal immune cell expression of TIM‐3 (HR 0.60, 95% CI 0.42–0.84, *p* = 0.003; Figure 2E), LAG‐3 (HR 0.58, 95% CI 0.40–0.87, *p* = 0.006; Figure 2C), and PD‐1 (HR 0.65, 95% CI 0.49–0.86, *p* = 0.002; Figure 2D) were significantly associated with improved cancer‐specific survival.

**Table 1:** Immune checkpoint expression in CRC patients and survival.

	Tumour			Stromal immune cells		
	*n* (%)	10 year CSS	*P* value	*n* (%)	10 year CSS	*P* value
TIM‐3	*n* = 401			*n* = 455		
Low	206	68%	0.188	186	59%	**0.003**
High	195	74%		271	75%	
LAG‐3	*n* = 412			*n* = 386		
Low	253	73%	**0.049**	196	63%	**0.006**
High	160	65%		191	78%	
PD‐1	*n* = 717			*n* = 714		
Low	494	72%	**0.047**	308	65%	**0.002**
High	223	66%		406	75%	
PD‐L1	*n* = 704			*n* = 703		
Low	452	71%	0.487	255	67%	0.298
High	252	68%		448	72%	

Bold values are less than 0.05.

### Immune checkpoint expression is associated with patient characteristics and clinicopathological parameters

Table 2 shows the relationship between prognostic immune checkpoints, patient characteristics, tumour characteristics, TME characteristics, and markers of systemic inflammation. As there were more than 10 comparisons performed, all analysis was corrected using the Bonferroni test. Therefore, associations were observed between high TIM‐3 expression on stromal immune cells and high Klintrup–Mäkinen grade (*p* < 0.001) and a low GMS (*p* < 0.001). Whereas, high tumoural PD‐1 associated with younger patients (*p* < 0.001), rectal tumours (*p* = 0.001), high TNM stage (*p* = 0.002), low Ki67 index (*p* < 0.001), high budding (*p* = 0.002), and unperforated tumours (*p* < 0.001). Finally, PD‐1 stromal immune cell expression associated with high Ki67 Index (*p* = 0.002), a high Klintrup–Mäkinen grade (*p* < 0.001), and low GMS (*p* < 0.001). No significant associations were seen for any other markers when corrected using the Bonferroni test.

**Table 2:** Relationship between immune checkpoint expression and clinicopathological characteristics in CRC patients

	TIM‐3 stromal immune cells			LAG‐3 tumour			LAG‐3 stromal immune cells			PD‐1 tumour			PD‐1 stromal immune cells		
	*n* = 457			*n* = 413			*n* = 387			*n* = 722			*n* = 719		
	Low *n* = 186 (41%)	High *n* = 271 (59%)	*P* value	Low *n* = 253 (61%)	High *n* = 160 (39%)	*P* value	Low *n* = 196 (51%)	High *n* = 191 (49%)	*P* value	Low *n* = 499 (69%)	High *n* = 223 (31%)	*P* value	Low *n* = 308 (43%)	High *n* = 411 (57%)	*P* value
Patient and tumour characteristics															
Age															
<65	53 (28)	99 (36)	0.074	77 (30)	54 (34)	0.481	57 (29)	70 (37)	0.113	133 (27)	91 (41)	**<0.001**	100 (32)	124(30)	0.511
>65	133 (72)	172 (64)		176 (70)	106 (66)		139 (71)	121 (63)		366 (73)	132 (59)		208 (68)	287 (70)	
Sex															
Male	82 (44)	147 (54)	**0.033**	125 (49)	85 (53)	0.461	96 (49)	95 (50)	0.881	254 (51)	117 (52)	0.698	159 (52)	211(51)	0.940
Female	104 (56)	124 (46)		128 (51)	75 (47)		100 (51)	96 (50)		245 (49)	106 (48)		149 (48)	200 (49)	
Site															
colon – right	84 (45)	121 (45)	0.832	98 (39)	74 (46)	0.319	79 (40)	79 (41)	0.970	219 (44)	90 (40)	**0.001**	132 (43)	176 (43)	0.962
Colon – left	63 (34)	87 (32)		91 (36)	51 (32)		69 (35)	67 (35)		182 (36)	62 (28)		105 (34)	137 (33)	
Rectum	39 (21)	63 (23)		64 (25)	35 (22)		48 (24)	45 (24)		98 (20)	71 (32)		71 (23)	98 (24)	
T‐stage															
1	2 (1)	15 (6)	**0.023**	10 (4)	7 (4)	0.161	6 (3)	11 (6)	0.322	25 (5)	9 (4)	**0.027**	13 (4)	21 (5)	0.115
2	25 (13)	38 (14)		44 (17)	16 (10)		24 (12)	30 (16)		73 (15)	16 (7)		28 (9)	61 (15)	
3	112 (60)	134 (49)		124 (49)	92 (58)		111 (57)	94 (49)		263 (53)	136 (61)		177 (57)	218 (53)	
4	47 (25)	84 (31)		74 (29)	45 (28)		55 (28)	56 (29)		138 (28)	62 (28)		90 (29)	111 (27)	
N‐stage										*n* = 720			*n* = 717		
0	107 (58)	172 (63)	**0.030**	159 (63)	92 (58)	0.172	119 (61)	117 (61)	0.770	326 (66)	127 (57)	**0.038**	179 (58)	273 (67)	0.058
1	50 (27)	78 (29)		60 (24)	51 (32)		51 (26)	53 (28)		118 (24)	73 (33)		91 (30)	98 (24)	
2	29 (16)	21 (8)		34 (13)	17 (11)		26 (13)	21 (11)		53 (11)	23 (10)		38 (12)	38 (9)	
TNM staging															
I	22 (12)	38 (14)	0.275	44 (17)	15 (9)	0.071	23 (12)	31 (16)	0.433	85 (17)	17 (8)	**0.002**	34 (11)	68 (17)	**0.025**
II	85 (46)	131 (48)		113 (45)	77 (48)		95 (48)	86 (45)		240 (48)	109 (49)		144 (47)	204 (50)	
III	79 (42)	102 (38)		96 (38)	68 (43)		78 (40)	74 (39)		174 (35)	97 (43)		130 (42)	139 (34)	
Ki67 index	*n* = 453			*n* = 408			*n* = 382			*n* = 717			*n* = 714		
<30	88 (47)	136 (51)	0.448	114 (46)	80 (51)	0.321	89 (46)	97 (51)	0.308	173 (35)	158 (72)	**<0.001**	161 (53)	170 (41)	**0.002**
>30	98 (53)	131 (49)		136 (54)	78 (49)		104 (54)	92 (49)		325 (65)	61 (28)		142 (47)	241 (59)	
Tumour differentiation															
Well	161 (87)	236 (87)	0.870	221 (87)	141 (88)	0.816	172 (88)	170 (89)	0.701	452 (91)	192 (86)	0.079	268 (87)	373 (91)	0.112
Poor	25 (13)	35 (13)		32 (13)	19 (12)		24 (12)	21 (11)		47 (9)	31 (14)		40 (13)	38 (9)	
Tumour budding	*n* = 407			*n* = 373			*n* = 347			*n* = 662			*n* = 660		
<25	112 (67)	169 (70)	0.473	168 (71)	95 (69)	0.707	130 (74)	117 (68)	0.263	359 (73)	103 (60)	**0.002**	189 (61)	272 (60)	0.596
>25 or more	55 (33)	71 (30)		68 (29)	42 (31)		46 (26)	54 (32)		132 (27)	68 (40)		86 (39)	113 (40)	
Tumour necrosis				*n* = 409			*n* = 383			*n* = 712			*n* = 709		
Low	117 (63)	177 (65)	0.597	159 (64)	97 (61)	0.510	121 (62)	116 (61)	0.841	303 (62)	127 (58)	0.332	185 (61)	243 (60)	0.818
High	69 (37)	94 (35)		90 (36)	63 (39)		73 (38)	73 (39)		189 (38)	93 (42)		119 (39)	162 (40)	
Tumour perforation	*n* = 433			*n* = 390			*n* = 387			*n* = 722			*n* = 676		
0	178 (96)	255 (94)	0.596	241 (95)	149 (93)	**0.017**	187 (95)	180 (94)	0.648	464 (93)	215 (96.4)	**<0.001**	291 (94.5)	385 (94)	0.854
1	1 (1)	4 (1)		0 (0)	5 (3)		3 (2)	2 (1)		2 (0.4)	7 (3.14)		4 (1.3)	5 (1)	
2	7 (4)	12 (4)		12 (5)	6 (4)		6 (3)	9 (5)		33 (6.6)	1 (0.45)		13 (4.2)	21 (5)	
Venous invasion															
Absent	116 (62)	183 (68)	0.255	166 (66)	109 (68)	0.597	128 (65)	134 (70)	0.307	346 (69)	143 (64)	0.168	192 (62)	293 (71)	**0.011**
Present	70 (38)	88 (32)		87 (34)	51 (32)		68 (35)	57 (30)		153 (31)	80 (36)		116 (38)	118 (29)	
Peritoneal involvement															
Absent	138 (74)	188 (69)	0.261	176 (70)	117 (73)	0.436	142 (72)	135 (71)	0.700	368 (74)	158 (71)	0.421	218 (71)	304 (74)	0.344
Involved	48 (26)	83 (31)		77 (30)	43 (27)		54 (28)	56 (29)		131 (26)	65 (29)		90 (29)	107 (26)	
Margin involvement															
Absent	174 (94)	255 (94)	0.811	240 (95)	150 (94)	0.633	184 (93)	181 (95)	0.707	473 (95)	209 (94)	0.566	286 (93)	393 (96)	0.112
Involved	12 (6)	16 (6)		13 (5)	10 (6)		12 (6)	10 (5)		26 (5)	14 (6)		22 (7)	18 (4)	
MMR status	*n* = 452			*n* = 407			*n* = 380			*n* = 714			*n* = 711		
Deficient	29 (16)	59 (22)	0.096	32 (13)	38 (24)	**0.005**	27 (14)	39 (21)	0.077	78 (16)	39 (18)	0.431	38 (13)	79 (19)	**0.015**
Competent	155 (84)	209 (78)		216 (87)	121 (76)		166 (86)	148 (79)		420 (84)	177 (82)		264 (87)	330 (81)	
TME characteristics															
TSP	*n* = 441			*n* = 400			*n* = 374			*n* = 706			*n* = 703		
Low	140 (77)	188 (72)	0.231	187 (76)	121 (78)	0.687	144 (76)	143 (77)	0.800	395 (79)	154 (74)	0.128	225 (74)	320 (80)	0.072
High	41 (23)	72 (28)		58 (24)	34 (22)		45 (24)	42 (23)		103 (21)	54 (26)		78 (26)	80 (20)	
K‐M score	*n* = 403			*n* = 409			*n* = 383			*n* = 714			*n* = 711		
Low‐grade	143 (77)	165 (61)	**<0.001**	167 (67)	104 (65)	0.666	142 (73)	111 (59)	**0.003**	329 (67)	150 (68)	0.854	230 (75)	247 (61)	**<0.001**
High‐grade	43 (23)	106 (39)		82 (33)	56 (35)		52 (27)	78 (41)		163 (33)	72 (32)		75 (25)	159 (39)	
GMS	*n* = 443			*n* = 398			*n* = 372			*n* = 701			*n* = 698		
0	42 (23)	106 (41)	**<0.001**	82 (34)	55 (35)	0.854	52 (28)	77 (42)	**0.006**	161 (33)	72 (34)	0.394	75 (25)	157 (39)	**<0.001**
1	105 (58)	108 (41)		117 (49)	78 (50)		106 (56)	75 (41)		260 (53)	100 (48)		161 (54)	196 (49)	
2	34 (19)	48 (18)		42 (17)	24 (15)		30 (16)	32 (17)		71 (14)	37 (18)		64 (21)	45 (11)	
Systemic inflammation															
CRP	*n* = 344			*n* = 320			*n* = 301			*n* = 580			*n* = 577		
Normal (≤10 mg/l)	78 (55)	108 (53)	0.698	114 (61)	65 (49)	**0.032**	99 (62)	69 (49)	**0.017**	195 (54)	122 (55)	0.835	141 (56)	173 (53)	0.457
Abnormal (>10 mg/l)	63 (45)	95 (47)		73 (39)	68 (51)		60 (38)	73 (51)		164 (46)	99 (45)		110 (44)	153 (47)	
Albumin	*n* = 361			*n* = 334			*n* = 314			*n* = 617			*n* = 614		
Abnormal (<35 g/l)	104 (71)	171 (80)	**0.046**	163 (83)	110 (80)	0.570	144 (87)	116 (78)	**0.050**	302 (76)	189 (85)	**0.009**	207 (77)	283 (82)	0.121
Normal (>35 g/l)	43 (29)	43 (20)		34 (17)	27 (20)		22 (13)	32 (22)		93 (24)	33 (15)		62 (23)	62 (18)	
mGPS	*n* = 347			*n* = 321			*n* = 302			*n* = 582			*n* = 579		
0	80 (56)	109 (53)	0.717	114 (61)	66 (50)	0.140	100 (63)	69 (49)	**0.039**	196 (54)	122 (55)	**0.024**	143 (57)	172 (53)	0.290
1	31 (22)	64 (31)		49 (26)	46 (35)		44 (28)	49 (35)		94 (26)	73 (33)		65 (26)	103 (32)	
2	31 (22)	32 (16)		25 (13)	21 (16)		16 (10)	24 (17)		71 (20)	26 (12)		45 (18)	51 (16)	
NLR	*n* = 348			*n* = 324			*n* = 304			*n* = 581			*n* = 578		
≤5	102 (71)	152 (74)	0.561	149 (76)	91 (71)	0.240	120 (75)	107 (75)	0.954	288 (74)	140 (73)	0.888	187 (75)	238 (72)	0.456
>5	41 (29)	53 (26)		46 (24)	38 (29)		41 (25)	36 (25)		102 (26)	51 (27)		62 (25)	91 (28)	

Bold values are less than 0.05.

### Immune checkpoint expression is associated with the immune landscape

Next, associations with the local immune lymphocytic infiltrate were assessed (see supplementary material, Table S1). High TIM‐3 stromal immune cell expression was significantly associated with high stromal CD3^+^ T‐cells (*p* < 0.001) and a total CD3^+^ high in both cancer cell nest and stroma score (*p* < 0.001), high CD8^+^ T‐cells in cancer cell nests (*p* = 0.001), high stromal CD8^+^ T‐cells (*p* < 0.001), a total CD8^+^ high in both cancer cell nest and stroma score (*p* < 0.001), high stromal FoxP3^+^ T‐cells (*p* < 0.001) and a total FoxP3^+^ high in both cancer cell nest and stroma score (*p* = 0.013). In contrast, tumoural LAG‐3 showed no significant relation with T‐cell markers. However, high LAG‐3 stromal immune cell expression associated with high CD3^+^ T‐cells in cancer cell nests (*p* = 0.024), high stromal CD3^+^ T‐cells (*p* = 0.001), a total CD3^+^ high in both cancer cell nest and stroma score (*p* = 0.002), high CD8^+^ T‐cells in cancer cell nest (*p* = 0.003), high stromal CD8^+^ T‐cells (*p* = 0.001) and a total CD8^+^ high in both cancer cell nest and stroma score (*p* = 0.002).

High tumoural PD‐1 was associated with low CD3^+^ T‐cells in cancer cell nests (*p* < 0.001), a total CD3^+^ low in both cancer cell nest and stroma score (*p* = 0.021), low CD8^+^ T‐cells in cancer cell nests (*p* = 0.001), low stromal CD8^+^ T‐cells (*p* < 0.001), and a total CD8^+^ low in both cancer cell nest and stroma score (*p* < 0.001), as well as low FoxP3^+^ T‐cells in cancer cell nests (*p* < 0.001) and a total FoxP3^+^ low in both cancer cell nest and stroma score (*p* < 0.001). Finally, high PD‐1 stromal immune cell expression associated with high CD3^+^ T‐cells in cancer cells nests (*p* < 0.001), high stromal CD3^+^ T‐cells (*p* < 0.001), a total CD3^+^ high in both cancer cell nest and stroma score (*p* < 0.001), high CD8^+^ T‐cells in cancer cell nests (*p* < 0.001), high stromal CD8^+^ T‐cells (*p* < 0.001), and a total CD8^+^ high in both cancer cell nest and stroma score (*p* < 0.001), as well as high FoxP3^+^ T‐cells in cancer cell nests (*p* < 0.001), high stromal FoxP3^+^ T‐cells (*p* < 0.001) and a total FoxP3^+^ high in both cancer cell nest and stroma score (*p* < 0.001).

### Combined immune checkpoint stromal score

**Figure 3:**
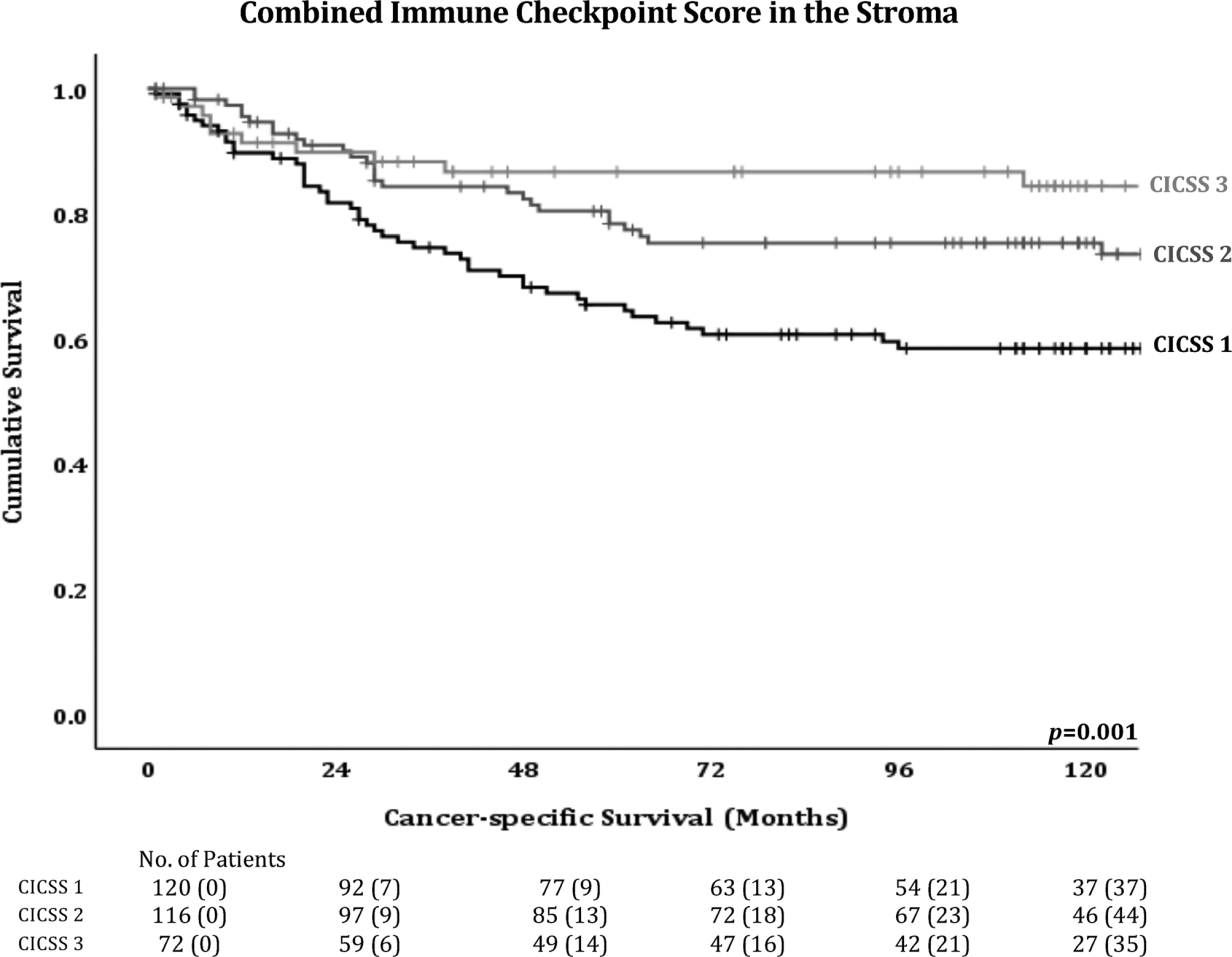
Combined Immune Checkpoint Stromal Score is significantly associated with survival. Kaplan–Meier survival curves for associations between CICSS and cancer‐specific survival.

Since expression on immune cells in the stroma of all three immune checkpoints displayed similar effects on patient prognosis, they were combined into a single score called the Combined Immune Checkpoint Stromal Score (CICSS) to assess if this increased their prognostic power. Only patients who had a valid score for all three markers were considered (*n* = 309). CICSS was defined as CICSS 3 = all 3 checkpoint markers high, CICSS 2 = 2 checkpoint markers high, and CICSS 1 = remaining patients. CICSS 3 significantly associated with improved cancer‐specific survival (HR 0.57, 95% CI 0.42–0.78, *p* = 0.001; Figure 3). The association between CICSS and clinicopathological characteristics was investigated (see supplementary material, Table S2). Again, as there were more than 10 comparisons, all analysis was corrected using the Bonferonni test. CICSS 3 was significantly associated with a high Klintrup–Mӓkinen grade (*p* < 0.001) and a low GMS (*p* = 0.001). Furthermore, when assessing the local inflammatory infiltrate (see supplementary material, Table S3), CICSS 3 was significantly associated with high CD3^+^ T‐cells (*p* < 0.001) and CD8^+^ T‐cells (*p* < 0.001) in cancer cell nests, high stromal CD3^+^ T‐cells (*p* < 0.001) and CD8^+^ T‐cells (*p* < 0.001) and a total CD3^+^ or CD8^+^ high in both cancer cell nest and stroma score (both *p* < 0.001), as well as high stromal FoxP3^+^ T‐cells (*p* = 0.001), and a total FoxP3^+^ high in both cancer cell nest and stroma score (*p* = 0.010).

### LAG‐3 stromal immune cell expression is an independent prognostic marker for CRC

Individual immune checkpoint markers and CICSS were entered in univariate cox regression analysis as shown in Table 3. TIM‐3 (*p* = 0.003), LAG‐3 (*p* = 0.007) and PD‐1 (*p* = 0.002) stromal immune cell expression, as well as tumoural PD‐1 (*p* = 0.049) and CICSS (*p* < 0.001) were significantly associated with cancer‐specific survival. These were taken forward into multivariate analysis with other prognostic clinical factors (Table 3), where TNM stage (*p* < 0.001), perforation (*p* = 0.001), GMS (*p* < 0.001), mGPS (*p* = 0.001) and LAG‐3 stromal immune cell expression (*p* = 0.017) were independently associated with cancer‐specific survival.

**Table 3:** Immune checkpoint expression, clinicopathological characteristics and cancer‐specific survival in CRC patients

	Univariate analysis		Multivariate analysis			
			Individual checkpoints		CICSS	
	HR (95% CI)	*P* value	HR (95% CI)	*P* value	HR (95% CI)	*P* value
Age (<65/>65 years)	1.03 (0.80–1.33)	0.815	–	–	–	–
Sex (male/female)	1.13 (0.88–1.44)	0.333	–	–	–	–
Tumour site (colon/rectum)	0.98 (0.84–1.14)	0.775	–	–	–	–
T‐stage (1/2/3/4)	1.94 (1.60–2.34)	**<0.001**	0.96 (0.34–2.73)	0.938	0.98 (0.35–2.73)	0.974
N‐stage (0/1/2)	2.06 (1.77–2.41)	**<0.001**	1.07 (0.53–2.16)	0.862	1.15 (0.57–2.32)	0.691
TNM stage (I/II/III)	2.48 (2.01–3.06)	**<0.001**	2.72 (1.59–4.66)	**<0.001**	2.76 (1.63–4.67)	**<0.001**
Ki67 index (low/high)	0.67 (0.53–0.86)	**0.001**	1.06 (0.49–2.28)	0.891	1.27 (0.67–2.43)	0.459
Tumour differentiation (well/poor)	2.02 (1.45–2.82)	**<0.001**	1.10 (0.50–2.44)	0.812	1.11 (0.51–2.43)	0.796
Tumour budding (low/high)	1.30 (1.00–1.71)	0.060	–	–	–	–
Tumour necrosis (low/high)	1.30 (1.01–1.66)	**0.040**	1.16 (0.63–2.12)	0.641	1.27 (0.71–2.27)	0.415
Tumour perforation (absent/present)	1.56 (1.27–1.92)	**<0.001**	2.66 (1.61–4.40)	**<0.001**	2.64 (1.56–4.36)	**<0.001**
Margin involvement (absent/involved)	3.44 (2.35–5.04)	**<0.001**	1.43 (0.62–3.28)	0.399	1.81 (0.84–3.90)	0.132
Venous invasion (absent/present)	2.14 (1.68–2.73)	**<0.001**	1.72 (0.98–3.02)	0.059	1.63 (0.92–2.88)	0.093
Peritoneal involvement (uninvolved/involved)	2.56 (2.01–3.27)	**<0.001**	1.03 (0.53–2.02)	0.921	0.92 (0.48–1.77)	0.795
MMR status (deficient/competent)	0.83 (0.59–1.16)	0.274	–	–	–	–
TSP (low/high)	1.95 (1.50–2.52)	**<0.001**	1.27 (0.22–7.175)	0.789	1.17 (0.21–6.60)	0.863
CD3 cancer cell nest (low/high)	0.56 (0.43–0.73)	**<0.001**	0.82 (0.41–1.64)	0.572	1.02 (0.50–2.06)	0.960
CD3 stroma (low/high)	0.59 (0.45–0.76)	**<0.001**	0.613 (0.35–1.08)	0.093	0.51 (0.29–0.88)	**0.016**
K‐M score (low‐grade/high‐grade)	0.40 (0.29–0.55)	**<0.001**	0.50 (0.16–1.59)	0.240	0.44 (0.14–1.38)	0.160
GMS (0/1/2)	1.89 (1.58–2.26)	**<0.001**	2.20 (1.46–3.32)	**<0.001**	2.00 (1.34–2.99)	**0.001**
CRP (normal/high)	2.09 (1.60–2.73)	**<0.001**	0.60 (0.05–7.88)	0.699	0.32 (0.03–3.62)	0.358
Albumin (low/normal)	2.13 (1.61–2.82)	**<0.001**	0.687 (0.28–1.71)	0.421	0.87 (0.37–2.04)	0.751
mGPS (0/1/2)	1.77 (1.50–2.08)	**<0.001**	1.91 (1.31–2.77)	**0.001**	1.82 (1.25–2.67)	**0.002**
NLR (low/high)	1.43 (1.08–1.91)	**0.013**	0.74 (0.35–1.57)	0.436	0.86 (0.41–1.81)	0.691
Immune checkpoint expression						
TIM‐3 tumour (low/high)	0.78 (0.53–1.14)	0.190	–	–	–	–
TIM‐3 stromal immune cells (low/high)	0.60 (0.42–0.84)	**0.003**	0.75 (0.43–1.30)	0.306	–	–
LAG‐3 tumour (low/high)	1.45 (1.00–2.09)	0.051	–	–	–	–
LAG‐3 stromal immune cells (low/high)	0.58 (0.40–0.87)	**0.007**	0.50 (0.28–0.88)	**0.017**	–	–
PD‐1 tumour (low/high)	1.34 (1.00–1.78)	**0.049**	0.810 (0.42–1.55)	0.523	–	–
PD‐1 stromal immune cells (low/high)	0.65 (0.49–0.86)	**0.002**	1.48 (0.84–2.61)	0.172	–	–
CICSS (1/2/3)	0.57 (0.42–0.78)	**<0.001**	–	–	0.856 (0.55–1.33)	0.486

## Discussion

This retrospective study on the prognostic value of the immune checkpoints TIM‐3, LAG‐3, PD‐1 and ligand PD‐L1 highlights the importance of the location and level of immune checkpoint expression in terms of CRC prognosis. Of the immune checkpoints investigated, high expression in the tumour was associated with decreased cancer‐specific survival, while on immune cells within the stroma it was associated with improved cancer‐specific survival. Furthermore, it supports the hypothesis that there is increased value gained from investigating multiple checkpoint markers as patients with stromal immune cell expression of all three checkpoints had the best prognosis.

To our knowledge, this is the first study to report that LAG‐3 and PD‐1 are expressed in CRC tumour cells. High PD‐1 in the tumour was associated with poorer survival and associated with poor prognostic factors including low proliferation index [37]. This suggests a possible role of PD‐1 in tumour progression. Furthermore, the positive association observed between high tumoural PD‐1 and low albumin [38] suggests a link between tumoural PD‐1 and systemic inflammation. Moreover, high PD‐1 in the tumour associated with low CD3^+^, CD8^+^ and FoxP3^+^ T‐cells, which are also associated with poorer prognosis. However, since this is the first study to report the expression of LAG‐3 and PD‐1 in tumour cells, these results require further validation. Since PD‐1's ligand PD‐L1 was not associated with survival, this study focussed on the immune checkpoint receptors' prognostic value.

In contrast, it was observed that high immune checkpoint expression on immune cells within the stroma associated with improved cancer‐specific survival and good prognostic factors. High PD‐1 stromal immune cell expression was shown to associate with early TNM stage and high Ki67 index [37] and could infer a role in regulation of growth and survival processes. Moreover, high PD‐1 stromal immune cell expression was associated with both high Klintrup–Mӓkinen grade and low GMS, as well as high densities of markers of adaptive immunity (CD3, CD8 and FoxP3). High Klintrup–Mӓkinen grade and low GMS have both been associated with improved survival, with Klintrup–Mӓkinen being associated with increased levels of CD3, CD8 and FoxP3 and reduced cancer recurrence, propagating a positive association [31, 39]. Hence, the results suggest that high PD‐1 stromal immune cell expression is a possible marker of the host's anti‐tumour immune response.

As with PD‐1 stromal immune cell expression, high levels of TIM‐3 and LAG‐3 stromal immune cell expression were individually associated with improved outcomes and markers of different T‐cell subsets. CD3^+^ and CD8^+^ cells are associated with a favourable outcome [40] and FoxP3^+^ cells, although a marker of the immunosuppressive regulatory T‐cells (Tregs), have also been reported to associate with better patient survival in CRC [41]. This further supports the relation between expression of these immune checkpoints in the stroma and improved patient survival due to effects on the local lymphocytic infiltrate. However, only LAG‐3 stromal immune cell expression was an independent prognostic factor, suggesting the other two checkpoints may rely on LAG‐3 for their function or expression.

Since the effects of high expression of each immune checkpoint on immune cells within the stroma had similar effects on patient survival, they were combined into a single score, CICSS, to investigate whether they might work together to improve prognostic power. A CICSS 3, where all three immune checkpoints are highly expressed, significantly improved patient survival. This highlights the importance of investigating the effects of immune checkpoints collectively rather than individually as the prognostic power was increased in the combined score compared to the individual proteins. CICSS 3 also associated with inflammatory factors of good prognosis such as high Klintrup–Mäkinen grade, and low GMS, as well as high levels of CD3^+^ T‐cells, CD8^+^cytotoxic T‐cells and FoxP3^+^ Tregs. Taken together, these results suggest that the combined high expression of these immune checkpoints in the stroma is a marker of the host's anti‐tumour immune response during the early stages of cancer development. However, CICSS was not an independent prognostic factor, but this may be due to low patient numbers for this analysis and needs validation in a larger independent cohort, which would also serve to validate the cut‐offs.

These results are contrary to the current approach towards these inhibitory checkpoints, which tends to look at them as facilitators of tumour progression. Of course, this approach is backed by functional studies where blockage or inhibition of these checkpoints led to tumour regression in many types of cancer such as melanoma, non‐small cell lung cancer, and even in metastatic CRC [42, 43, 44]. However, it is important to note the differences between the methodology used here and in previous work. For instance, recent studies have primarily focused on circulating immune cells, tumour cells, cell lines, or animal models [45, 46, 47, 48, 49], and few of them utilise CRC patient tissue or include all three immune checkpoints [50, 51, 52, 53].

To our knowledge, this paper is the first to investigate these three immune checkpoints together in the context of the human CRC TME and prognosis. These findings could not only serve as a basis for including multiple immune checkpoints in future prognostic studies on CRC but could also be useful in clinical trials. The adaptation of such prognostic information may lead to the development of statistical models that could predict the outcome of therapies in patients in the same immune checkpoint expression subset. Further functional analysis of these results is warranted, and any underlying mechanisms must be elucidated. The correlation between each of these checkpoints and their tumour‐secreted ligands would be interesting to explore, as well as relating each immune checkpoint to the T‐cell subset it is most highly expressed in, and whether there are any underlying associations with common mutations such as *BRAF*.

In conclusion, these results suggest that high levels of the inhibitory immune checkpoints TIM‐3, LAG‐3 and PD‐1, whether individually or in combination, on immune cells within the stroma are associated with good prognosis and it was observed that stromal LAG‐3 is an independent prognostic factor. Finally, the results point to the inclusion of all three immune checkpoints in future investigation regarding CRC prognosis.

## Author contributions statement

SSFA and LG performed experiments, scored TMAs, analysed data, and wrote the manuscript. MVC performed experiments and scored TMAs. JI performed immune experiments. KP and JQ aided in double scoring TMAs. PK is a consultant pathologist advising on all pathology and TMA construction. LH aided in cell pellet work. PGH aided in sample acquisition and manuscript editing. DCM, CSDR and JHP double scored, conceived the study, and aided in manuscript editing. AR double scored TMAs and aided in manuscript writing. JE conceived the study, double scored, analysed data and aided in manuscript writing and editing. All authors had final approval of the submitted version.

## Supporting information


**Figure S1.** Immunohistochemistry controls for TIM‐3, LAG‐3, PD‐1 and PD‐L1
**Figure S2.** CONSORT diagram of patient inclusion in the study

**Table S1.** Relationship between immune checkpoint expression and the immune landscape of CRC patients (T‐cell markers)
**Table S2.** Relationship between Combined Immune Checkpoint Score (CICSS) in the stroma and clinicopathological characteristics
**Table S3.** Relationship between Combined Immune Checkpoint Score in the stroma and the immune landscape
**Table S4.** Clinicopathological characteristics summary of entire CRC cohort
