# Supplementary material for: Relationship between immune checkpoint proteins, tumour microenvironment characteristics, and prognosis in primary operable colorectal cancer
Source: J Pathol Clin Res. 2020 Dec 18;7(2):121–34. doi: 10.1002/cjp2.193 (PMC7869939; doi:10.1002/cjp2.193)
Supplement: Supplementary file 1 — Figure S1. Immunohistochemistry controls for TIM‐3, LAG‐3, PD‐1 and PD‐L1 Figure S2. CONSORT diagram of patient inclusion in the study [file CJP2-7-121-s001.docx]

**Relationship between immune checkpoint proteins, tumour microenvironment characteristics, and prognosis in primary operable colorectal cancer**

Al-Badran SSF et al. J Pathol Clin Res DOI: 10.1002/cjp2.193

**Supplementary Figures**

**Figure S1. IHC Controls.** TIM-3: Large image of whole slide positive colorectal cancer tissue with higher magnification in the bottom right corner. Top right: negative colorectal cancer tissue. Middle right: positive liver tissue. Bottom right: positive HT29 colorectal cell pellets and negative liver isotype control, respectively. LAG-3: Large image of whole slide positive colorectal cancer tissue with higher magnification in the bottom right. Top right: negative colorectal cancer tissue. Middle right: positive liver tissue. Bottom right: negative HT29 colorectal cell pellets and negative liver isotype control, respectively. PD-1: Large image of whole slide positive colorectal cancer tissue with higher magnification in the bottom right. Top right: negative colorectal cancer tissue. Middle right: positive tonsil tissue. Bottom right: negative liver isotype control. PD-L1: Large image of whole slide positive colorectal cancer tissue with higher magnification in the bottom right. Top right: negative colorectal cancer tissue. Middle right: positive tonsil tissue. Bottom right: negative HT29 colorectal cell pellets and negative liver isotype control, respectively. Scale bar of large image 500μm and of small images 100μm.

**Figure S2.** CONSORT diagram of patient inclusion in the study
