# Supplementary material for: Relationship between immune checkpoint proteins, tumour microenvironment characteristics, and prognosis in primary operable colorectal cancer
Source: J Pathol Clin Res. 2020 Dec 18;7(2):121–34. doi: 10.1002/cjp2.193 (PMC7869939; doi:10.1002/cjp2.193)
Supplement: Supplementary file 2 — Table S1. Relationship between immune checkpoint expression and the immune landscape of CRC patients (T‐cell markers) Table S2. Relationship between Combined Immune Checkpoint Score (CICSS) in the stroma and clinicopathological characteristics Table S3. Relationship between Combined Immune Checkpoint Score in the stroma and the immune landscape Table S4. Clinicopathological characteristics summary of entire CRC cohort [file CJP2-7-121-s002.docx]

**Relationship between immune checkpoint proteins, tumour microenvironment characteristics, and prognosis in primary operable colorectal cancer**

Al-Badran SSF et al. J Pathol Clin Res DOI: 10.1002/cjp2.193

**Table S1.** Relationship between immune checkpoint expression and the immune landscape of CRC patients (T-cell markers)

|  | **TIM-3 Stromal Immune Cells** | | | **LAG-3 Tumour** | | | **LAG-3 Stromal Immune Cells** | | | **PD-1 Tumour** | | | **PD-1 Stromal Immune Cells** | | |
| --- | --- | --- | --- | --- | --- | --- | --- | --- | --- | --- | --- | --- | --- | --- | --- |
|  | **n=457** | | | **n=413** | | | **n=387** | | | **n=722** | | | **n=719** | | |
|  | **Low**  **n=186**  **(41%)** | **High**  **n=271**  **(59%)** | ***p*** | **Low**  **n=253**  **(61%)** | **High**  **n=160**  **(39%)** | ***p*** | **Low**  **n=196 (51%)** | **High**  **n=191**  **(49%)** | ***p*** | **Low**  **n=499**  **(69%)** | **High n=223**  **(31%)** | ***p*** | **Low n=308**  **(43%)** | **High n=411**  **(57%)** | ***p*** |
| **CD3 Cancer cell nest** | **n=449** | | | **n=403** | | | **n=376** | | | **n=710** | | | **n=707** | | |
| Low | 105 (58) | 132 (49) | 0.068 | 123 (50) | 84 (53) | 0.561 | 110 (58) | 85 (46) | **0.024** | 236 (48) | 139 (64) | **<0.001** | 212 (70) | 163 (40) | **<0.001** |
| High | 76 (42) | 136 (51) |  | 122 (50) | 74 (47) |  | 81 (42) | 100 (54) |  | 257 (52) | 78 (36) |  | 91 (30) | 241 (60) |  |
| **CD3 Stroma** | **n=449** | | | **n=403** | | | **n=376** | | | **n=710** | | | **n=707** | | |
| Low | 110 (61) | 99 (37) | **<0.001** | 109 (44) | 73 (46) | 0.736 | 102 (53) | 68 (37) | **0.001** | 230 (47) | 104 (48) | 0.754 | 179 (59) | 155 (38) | **<0.001** |
| High | 71 (39) | 169 (63) |  | 136 (56) | 85 (54) |  | 89 (47) | 117 (63) |  | 263 (53) | 113 (52) |  | 124 (41) | 249 (62) |  |
| **CD3 Total** | **n=449** | | | **n=403** | | | **n=376** | | | **n=710** | | | **n=707** | | |
| Both Low | 82 (45) | 80 (30) | **<0.001** | 82 (33) | 56 (35) | 0.855 | 75 (39) | 52 (28) | **0.002** | 158 (32) | 93 (43) | **0.021** | 154 (51) | 97 (24) | **<0.001** |
| One High | 51 (28) | 71 (26) |  | 68 (28) | 45 (28) |  | 62 (32) | 49 (26) |  | 150 (30) | 57 (26) |  | 83 (27) | 124 (31) |  |
| Both High | 48 (27) | 117 (44) |  | 95 (39) | 57 (36) |  | 54 (28) | 84 (45) |  | 185 (38) | 67 (31) |  | 66 (22) | 183 (45) |  |
| **CD8 Cancer Cell Nest** | **n=453** | | | **n=404** | | | **n=379** | | | **n=704** | | | **n=701** | | |
| Low | 122 (66) | 136 (51) | **0.001** | 145 (59) | 90 (57) | 0.784 | 124 (65) | 94 (50) | **0.003** | 269 (55) | 147 (68) | **0.001** | 235 (78) | 180 (45) | **<0.001** |
| High | 63 (34) | 132 (49) |  | 102 (41) | 67 (43) |  | 67 (35) | 94 (50) |  | 219 (45) | 69 (32) |  | 67 (22) | 219 (55) |  |
| **CD8 Stroma** | **n=452** | | | **n=403** | | | **n=378** | | | **n=703** | | | **n=700** | | |
| Low | 133 (72) | 132 (49) | **<0.001** | 137 (56) | 98 (62) | 0.181 | 126 (66) | 93 (50) | **0.001** | 267 (55) | 154 (72) | **<0.001** | 219 (73) | 202 (51) | **<0.001** |
| High | 52 (28) | 135 (51) |  | 109 (44) | 59 (38) |  | 65 (35) | 94 (50) |  | 221 (45) | 61 (28) |  | 83 (27) | 196 (49) |  |
| **CD8 Total** | **n=452** | | | **n=403** | | | **n=378** | | | **n=703** | | | **n=700** | | |
| Both Low | 105 (57) | 98 (37) | **<0.001** | 109 (44) | 75 (48) | 0.792 | 103 (54) | 70 (37) | **0.002** | 199 (41) | 125 (58) | **<0.001** | 193 (64) | 131 (33) | **<0.001** |
| One High | 45 (24) | 72 (27) |  | 64 (26) | 38 (24) |  | 44 (23) | 47 (25) |  | 138 (28) | 51 (24) |  | 68 (23) | 120 (30) |  |
| Both High | 35 (19) | 97 (36) |  | 73 (30) | 44 (28) |  | 44 (23) | 70 (37) |  | 151 (31) | 39 (18) |  | 41 (14) | 147 (37) |  |
| **FoxP3 Cancer Cell Nest** | **n=442** | | | **n=394** | | | **n=369** | | | **n=649** | | | **n=647** | | |
| Low | 67 (37) | 105 (40) | 0.545 | 79 (33) | 60 (39) | 0.194 | 70 (38) | 62 (33) | 0.288 | 154 (35) | 105 (50) | **<0.001** | 133 (48) | 126 (34) | **<0.001** |
| High | 113 (63) | 157 (60) |  | 162 (67) | 93 (61) |  | 112 (62) | 125 (67) |  | 284 (65) | 106 (50) |  | 144 (52) | 244 (66) |  |
| **FoxP3 Stroma** | **n=443** | | | **n=395** | | | **n=370** | | | **n=650** | | | **n=648** | | |
| Low | 134 (74) | 149 (57) | **<0.001** | 158 (66) | 98 (64) | 0.696 | 124 (68) | 111 (59) | 0.093 | 288 (66) | 127 (60) | 0.147 | 202 (73) | 213 (58) | **<0.001** |
| High | 47 (26) | 113 (43) |  | 83 (34) | 56 (36) |  | 59 (32) | 76 (41) |  | 150 (34) | 85 (40) |  | 76 (27) | 157 (42) |  |
| **FoxP3 Total** | **n=442** | | | **n=394** | | | **n=369** | | | **n=649** | | | **n=647** | | |
| Both Low | 59 (33) | 81 (31) | **0.013** | 68 (28) | 51 (33) | 0.433 | 63 (35) | 49 (26) | 0.203 | 121 (28) | 97 (46) | **<0.001** | 119 (43) | 99 (27) | **<0.001** |
| One High | 82 (46) | 92 (35) |  | 101 (42) | 55 (36) |  | 67 (37) | 75 (40) |  | 200 (46) | 37 (18) |  | 96 (35) | 141 (38) |  |
| Both High | 39 (22) | 89 (34) |  | 72 (30) | 47 (31) |  | 52 (29) | 63 (34) |  | 117 (27) | 77 (36) |  | 62 (22) | 130 (35) |  |

**Table S2.** Relationship between Combined Immune Checkpoint Score (CICSS) in the stroma and clinicopathological characteristics in CRC patients (n=309)

|  | **CICSS 1**  **n= 120 (39%)** | **CICSS 2**  **n=117 (38%)** | **CICSS 3**  **n= 72 (23%)** | ***p*** |
| --- | --- | --- | --- | --- |
| **Patient and Tumour Characteristics** | | | | |
| **Age** | | | | |
| <65 | 33 (28) | 44 (38) | 22 (31) | 0.490 |
| >65 | 87 (73) | 73 (62) | 50 (69) |  |
| **Sex** | | | | |
| Male | 58 (48) | 56 (48) | 40 (56) | 0.539 |
| Female | 62 (52) | 61 (52) | 32 (44) |  |
| **Site** | | | | |
| Colon - Right | 52 (43) | 49 (42) | 32 (44) | 0.390 |
| Colon - Left | 43 (36) | 34 (29) | 27 (38) |  |
| Rectum | 25 (21) | 34 (29) | 13 (18) |  |
| **T-Stage** | | | | |
| 1 | 4 (3) | 3 (3) | 7 (10) | **0.017** |
| 2 | 10 (8) | 23 (20) | 8 (11) |  |
| 3 | 73 (61) | 56 (48) | 37 (51) |  |
| 4 | 33 (28) | 36 (31) | 20 (28) |  |
| **N-Stage** | | | | |
| 0 | 72 (60) | 69 (59) | 49 (68) | 0.334 |
| 1 | 33 (28) | 35 (30) | 16 (22) |  |
| 2 | 15 (13) | 13 (11) | 7 (10) |  |
| **ΤΝΜ Staging** | | | | |
| I | 11 (9) | 16 (14) | 13 (18) | 0.111 |
| II | 61 (51) | 52 (44) | 36 (50) |  |
| III | 48 (40) | 49 (42) | 23 (32) |  |
| **Ki67 Index** | | | | |
| <30 | 61 (52) | 63 (54) | 30 (42) | 0.224 |
| >30 | 57 (48) | 53 (46) | 42 (58) |  |
| **Tumour Differentiation** | | | | |
| Well | 103 (86) | 104 (89) | 61 (85) | 0.663 |
| Poor | 17 (14) | 13 (11) | 11 (15) |  |
| **Tumour Budding** | | | | |
| <25 | 76 (71) | 72 (69) | 48 (71) | 0.920 |
| >25 or more | 31 (29) | 33 (31) | 20 (29) |  |
| **Tumour Necrosis** | | | | |
| Low | 71 (59) | 81 (69) | 39 (54) | 0.086 |
| High | 49 (41) | 36 (31) | 33 (46) |  |
| **Tumour Perforation** | | | | |
| 0 | 112 (93) | 114 (97) | 65 (90) | 0.345 |
| 1 | 2 (2) | 1 (1) | 2 (3) |  |
| 2 | 6 (5) | 2 (2) | 5 (7) |  |
| **Venous Invasion** | | | | |
| Absent | 77 (64) | 80 (68) | 55 (76) | 0.201 |
| Present | 43 (36) | 37 (32) | 17 (24) |  |
| **Peritoneal Involvement** | | | | |
| Absent | 87 (73) | 80 (68) | 52 (72) | 0.753 |
| Involved | 33 (28) | 37 (32) | 20 (28) |  |
| **Margin Involvement** | | | | |
| Absent | 111 (93) | 109 (93) | 71 (99) | 0.109 |
| Involved | 9 (8) | 8 (7) | 1 (1) |  |
| **MMR Status (n=306)** | | | | |
| Deficient | 14 (12) | 14 (12) | 20 (28) | **0.009** |
| Competent | 105 (88) | 101 (88) | 52 (72) |  |
| **TME Characteristics** | | | | |
| **TSP** | | | | |
| Low | 89 (77) | 81 (70) | 55 (81) | 0.212 |
| High | 27 (23) | 35 (30) | 13 (19) |  |
| **K-M Score** | | | | |
| Low-grade | 95 (79) | 77 (66) | 36 (50) | **<0.001** |
| High-grade | 25 (21) | 40 (34) | 36 (50) |  |
| **GMS** | | | | |
| 0 | 25 (22) | 39 (31) | 36 (52) | **0.001** |
| 1 | 67 (58) | 52 (41) | 25 (36) |  |
| 2 | 23 (20) | 36 (28) | 8 (12) |  |
| **Systemic Inflammation** | | | | |
| **CRP** | | | | |
| ≤10mg/L | 52 (57) | 52 (59) | 23 (45) | 0.260 |
| >10mg/L | 40 (43) | 36 (41) | 28 (55) |  |
| **Albumin** | | | | |
| <35g/L | 81 (83) | 71 (79) | 42 (79) | 0.780 |
| >35g/L | 17 (17) | 19 (21) | 11 (21) |  |
| **mGPS** | | | | |
| 0 | 53 (57) | 52 (59) | 23 (45) | 0.524 |
| 1 | 27 (29) | 23 (26) | 20 (39) |  |
| 2 | 13 (14) | 13 (15) | 8 (16) |  |
| **NLR** | | | | |
| ≤5 | 71 (76) | 66 (78) | 32 (63) | 0.150 |
| >5 | 23 (24) | 19 (22) | 19 (37) |  |
| Abbreviations: MMR = Mismatch Repair, TSP = Tumour Stroma Percentage, K-M Score = Klintrup-Mäkinen Score, GMS = Glasgow Microenvironment Score, CRP = C-Reactive Protein, mGPS = modified Glasgow Prognostic Score, NLR = Neutrophil:Lymphocyte Ratio | | | | |

**Table S3.** Relationship between Combined Immune Checkpoint Score in the stroma and the immune landscape in CRC Patients (n=309)

|  | **CICSS 1**  **n= 120** | **CICSS 2**  **n=117** | | **CICSS 3**  **n= 72** | ***p*** |
| --- | --- | --- | --- | --- | --- |
| **T-Cell Markers** | | | | | |
| **CD3 Cancer Cell Nest** | **n=118** | | **n=116** |  |  |
| Low | 81 (69) | 58 (50) | | 24 (33) | **<0.001** |
| High | 37 (31) | 58 (50) | | 48(67) |  |
| **CD3 Stroma** | **n=118** | | **n=116** |  |  |
| Low | 72 (61) | 44 (38) | | 20 (28) | **<0.001** |
| High | 46 (39) | 72 (62) | | 52 (72) |  |
| **CD3 Total** | **n=118** | | **n=116** |  |  |
| Both Low | 60 (51) | 31 (27) | | 14 (19) | **<0.001** |
| One High | 33 (28) | 40 (34) | | 16 (22) |  |
| Both High | 25 (21) | 45 (39) | | 42 (58) |  |
| **CD8 Cancer Cell Nest** | **n=118** | |  |  |  |
| Low | 87 (74) | 68 (58) | | 20 (28) | **<0.001** |
| High | 31 (26) | 49 (42) | | 52 (72) |  |
| **CD8 Stroma** | **n=118** | |  | **n=71** |  |
| Low | 89 (75) | 67 (57) | | 21 (30) | **<0.001** |
| High | 29 (25) | 50 (43) | | 50 (70) |  |
| **CD8 Total** | **n=118** | |  | **n=71** |  |
| Both Low | 78 (66) | 46 (39) | | 13 (18) | **<0.001** |
| One High | 20 (17) | 43 (37) | | 15 (21) |  |
| Both High | 20 (17) | 28 (24) | | 43 (61) |  |
| **FoxP3 Cancer Cell Nest** | **n=117** | |  |  |  |
| Low | 47 (40) | 43 (37) | | 18 (25) | 0.089 |
| High | 70 (60) | 74 (63) | | 54 (75) |  |
| **FoxP3 Stroma** | **n=118** | |  |  |  |
| Low | 88 (75) | 70 (60) | | 35 (49) | **0.001** |
| High | 30 (25) | 47 (40) | | 37 (51) |  |
| **FoxP3 Total** | **n=117** | |  |  |  |
| Both Low | 44 (38) | 32 (27) | | 14 (19) | **0.010** |
| One High | 46 (39) | 49 (42) | | 25 (35) |  |
| Both High | 27 (23) | 36 (31) | | 33 (46) |  |

**Table S4.** Clinicopathological characteristics summary of entire CRC cohort (n=773)

|  | **Patients**  **(%)** |
| --- | --- |
| **Patient and Tumour Characteristics** | |
| **Age** | |
| <65 | 245 (32) |
| >65 | 528 (68) |
| **Sex** | |
| Male | 395 (51) |
| Female | 378 (49) |
| **Site** | |
| Colon - Right | 330 (43) |
| Colon - Left | 258 (33) |
| Rectum | 185 (24) |
| **T-Stage** | |
| 1 | 34 (4) |
| 2 | 95 (12) |
| 3 | 426 (55) |
| 4 | 218 (28) |
| **N-Stage (n=771)** | |
| 0 | 482 (63) |
| 1 | 204 (26) |
| 2 | 85 (11) |
| **ΤΝΜ Staging** | |
| I | 107 (14) |
| II | 373 (48) |
| III | 293 (38) |
| **Ki67 Index (n=766)** | |
| <30 | 365 (48) |
| >30 | 401 (52) |
| **Tumour Differentiation** | |
| Well | 688 (89) |
| Poor | 85 (11) |
| **Tumour Budding (n=701)** | |
| <25 | 493 (70) |
| >25 or more | 208 (30) |
| **Tumour Necrosis (n=762)** | |
| Low | 465 (61) |
| High | 297 (39) |
| **Tumour Perforation** | |
| 0 | 729 (94) |
| 1 | 9 (1) |
| 2 | 35 (5) |
| **Venous Invasion** | |
| Absent | 516 (67) |
| Present | 257 (33) |
| **Peritoneal Involvement** | |
| Absent | 560 (72) |
| Involved | 213 (28) |
| **Margin Involvement** | |
| Absent | 727 (94) |
| Involved | 46 (6) |
| **MMR Status (n=761)** | |
| Deficient | 133 (17) |
| Competent | 628 (83) |
| **TME Characteristics** | |
| **TSP (n=751)** | |
| Low | 580 (77) |
| High | 171 (23) |
| **K-M Score (n=764)** | |
| Low-grade | 507 (66) |
| High-grade | 257 (34) |
| **GMS (n=747)** | |
| 0 | 255 (34) |
| 1 | 375 (50) |
| 2 | 117 (16) |
| **Systemic Inflammation** | |
| **CRP (n=621)** | |
| ≤10mg/L | 338 (54) |
| >10mg/L | 283 (46) |
| **Albumin (n=660)** | |
| <35g/L | 527 (80) |
| >35g/L | 133 (20) |
| **mGPS (n=624)** | |
| 0 | 340 (54) |
| 1 | 182 (29) |
| 2 | 102 (16) |
| **NLR (n=624)** | |
| ≤5 | 461 (74) |
| >5 | 163 (26) |
| Abbreviations: MMR = Mismatch Repair, TSP = Tumour Stroma Percentage, K-M Score = Klintrup-Mäkinen Score, GMS = Glasgow Microenvironment Score, CRP = C-Reactive Protein, mGPS = modified Glasgow Prognostic Score, NLR = Neutrophil:Lymphocyte Ratio | |
